# *In silico* models for evaluating proarrhythmic risk of drugs

**DOI:** 10.1063/1.5132618

**Published:** 2020-06-04

**Authors:** Minki Hwang, Chul-Hyun Lim, Chae Hun Leem, Eun Bo Shim

**Affiliations:** 1 SiliconSapiens Inc., Seoul 06097, South Korea; 2 Department of Mechanical and Biomedical Engineering, Kangwon National University, Chuncheon 24341, South Korea; 3 Department of Physiology, College of Medicine, University of Ulsan, Asan Medical Center, Seoul 05505, South Korea

## Abstract

Safety evaluation of drugs requires examination of the risk of generating Torsade de Pointes (TdP) because it can lead to sudden cardiac death. Until recently, the QT interval in the electrocardiogram (ECG) has been used in the evaluation of TdP risk because the QT interval is known to be associated with the development of TdP. Although TdP risk evaluation based on QT interval has been successful in removing drugs with TdP risk from the market, some safe drugs may have also been affected due to the low specificity of QT interval-based evaluation. For more accurate evaluation of drug safety, the comprehensive *in vitro* proarrhythmia assay (CiPA) has been proposed by regulatory agencies, industry, and academia. Although the CiPA initiative includes *in silico* evaluation of cellular action potential as a component, attempts to utilize *in silico* simulation in drug safety evaluation are expanding, even to simulating human ECG using biophysical three-dimensional models of the heart and torso under the effects of drugs. Here, we review recent developments in the use of *in silico* models for the evaluation of the proarrhythmic risk of drugs. We review the single cell, one-dimensional, two-dimensional, and three-dimensional models and their applications reported in the literature and discuss the possibility of utilizing ECG simulation in drug safety evaluation.

## I. INTRODUCTION

**FIG. 1.**
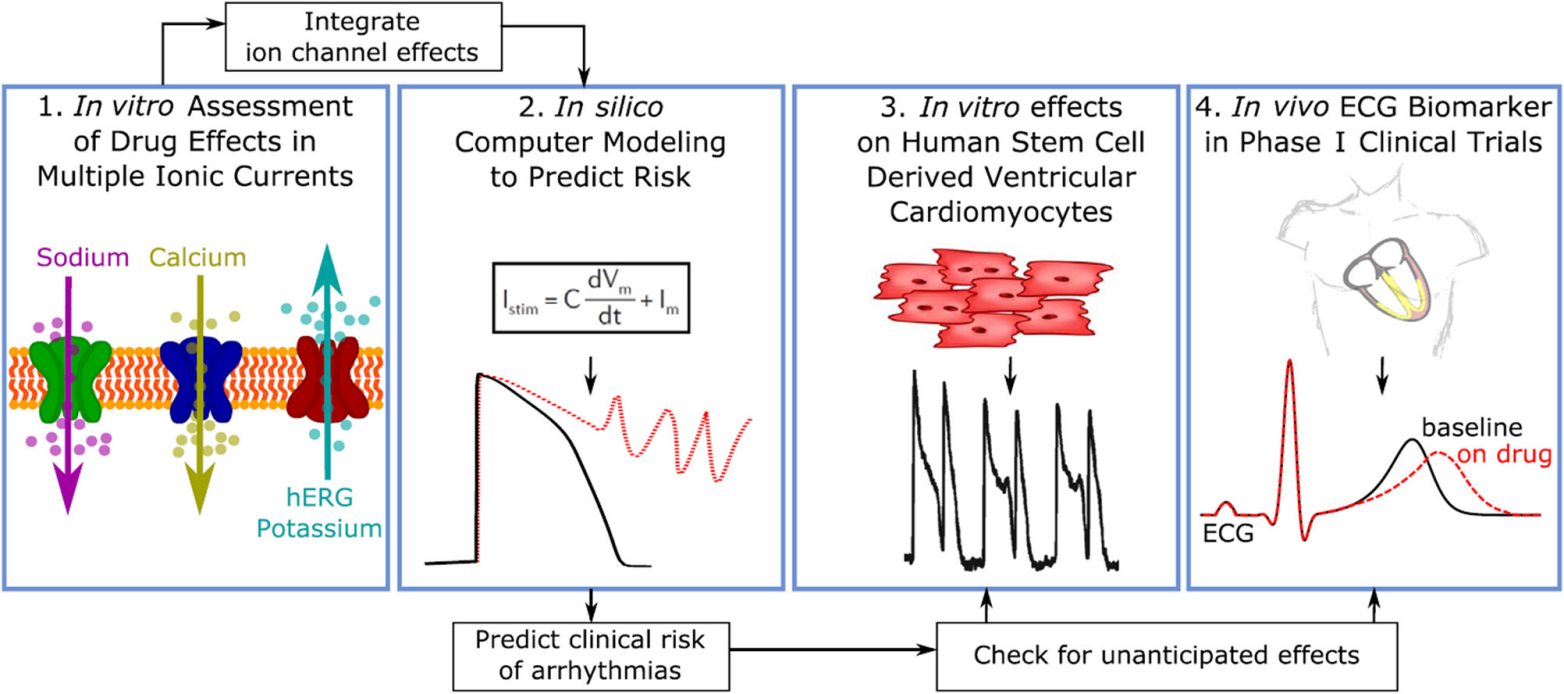
The four components of the comprehensive *in vitro* proarrhythmia assay. (1) Assessment of drug effects on multiple ion currents. (2) *In silico* prediction of cardiac action potential. (3) *In vitro* effects on human stem cell derived cardiac myocytes. (4) Human ECG in phase I clinical trials. Reproduced with permission from Vicente *et al.*, Clin. Pharmacol. Ther. **103**(1), 54–66 (2018) Copyright 2018 Authors, licensed under a Creative Commons Attribution Non-Commercial License.

The risk of generating Torsade de Pointes (TdP) must be examined in the safety evaluation of drugs because TdP can lead to sudden cardiac death. Until recently, the QT interval in the electrocardiogram (ECG) has been of primary interest in the evaluation of TdP risk because the QT interval is known to be associated with the development of TdP. Although TdP risk evaluation based on the QT interval has been successful in removing drugs with TdP risk from the market, some safe drugs may have also been affected due to the low specificity of QT interval-based evaluation.^1^ The QT interval is prolonged primarily due to blockade of the delayed rectifier potassium current (I_Kr_), but if the L-type calcium current (I_CaL_) and/or the late sodium current (I_NaL_) are also blocked, TdP risk is minimized even though QT prolongation is present.^2^ The comprehensive *in vitro* proarrhythmia assay (CiPA) has been proposed by regulatory agencies, industry, and academia for more accurate evaluation of drug safety.^3^ CiPA consists of the following four components: (1) assessment of drug effects on multiple ion currents, (2) *in silico* prediction of cardiac action potential (AP), (3) *in vitro* effects on human stem cell-derived cardiac myocytes, and (4) human ECG in phase I clinical trials (Fig. 1). The CiPA initiative led to the discovery of the JTpeak interval of ECG as a biomarker of TdP risk.^4^ The blockade of I_Kr_ in conjunction with I_CaL_ and/or I_NaL_, which showed antiarrhythmic effects, did not prolong the JTpeak interval despite a prolonged QT interval.^2^

Although *in silico* evaluation of cellular AP was included in the CiPA initiative as a component, attempts to utilize *in silico* simulation in drug safety evaluation are expanding, even to the simulation of human ECG using biophysical three-dimensional models of the heart and torso under the effects of drugs. In this article, we review recent developments in the use of *in silico* models for drug safety evaluation and discuss the possibility of utilizing ECG simulation in drug safety evaluation.

## II. SIMULATION METHODS FOR CARDIAC ELECTROPHYSIOLOGY

Cardiac myocytes are excitable cells in which the membrane potential increases rapidly when it exceeds a threshold. There are several models for the action potential of human cardiac myocytes. Ten Tusscher *et al.*^5^ (TT04) developed a mathematical model of the action potential of human ventricular cells based on experimental data on the major ionic currents. Their model reproduced the experimentally observed action potential duration (APD) restitution. They also updated their model to include a more extensive description of intracellular calcium dynamics (TT06).^6^ Grandi *et al.*^7^ developed a detailed mathematical model for calcium handling and ionic currents in human ventricular myocytes. They validated their model against experimental data, including APD adaptation and restitution. O'Hara *et al.*^8^ developed a human ventricular AP model (ORd model) using undiseased human ventricular data, including rate dependence and restitution of APD. Their model reproduced experiments for rate dependence of Ca^2+^ and intracellular sodium in undiseased human myocytes. The ORd model is the most updated model based on a large amount of human experimental data and has been selected as the starting point for developing an *in silico* model within the CiPA initiative.^1^ Although the ORd model has been widely used, it has some drawbacks, including high sensitivity to changes in I_Kr_.^9^ There have been attempts to optimize the baseline ORd model to increase its predictive power under various conditions. Mann *et al.*^10^ optimized three models of cellular electrophysiology, including the ORd model, by applying scaling factors to the maximum conductances for ionic currents. They obtained scaling factors by comparing the simulated change of APD_90_ with a clinically obtained change in the QT interval in patients with long QT syndrome (LQTS). The optimized model reproduced more accurately the prolongation of repolarization in all LQTS subtypes. Dutta *et al.*^11^ included a Markov model of I_Kr_ in the ORd model to represent dynamic interactions between drugs and I_Kr_. They also refined the model parameters using experimental data obtained in human cardiomyocytes under control and drug block conditions with the main purpose to improve model predictions of drug blocks. Krogh-Madsen *et al.*^9^ optimized the ORd model to clinical long QT data with the application of physiologically based bounds on intracellular calcium and sodium concentrations. They tested the optimized model against a database of known drugs and showed that it improved risk assessment. Asakura *et al.*^12^ developed a human ventricular cell model including the tight coupled L-type Ca^2+^ channel and ryanodine receptor (LCC–RyR) model based on control theory. They reproduced realistic Ca^2+^ dynamics and examined the Ca^2+^ mechanisms involved in the generation of early afterdepolarization (EAD) and delayed afterdepolarization (DAD) by applying the lead potential analysis. Recently, Tomek *et al.*^13^ developed a human-based ventricular model (ToR-ORd) based on the ORd model to overcome the inconsistencies presented by the currently available models. They performed calibration and validation of the model under healthy and key disease conditions as well as drug blockade.

The electrical signal propagation in the heart in the form of wave, which enables contraction of the heart, is simulated by solving reaction–diffusion equations numerically. The bidomain model consists of the following equations:^14^

$$\frac{\partial V_{m}}{\partial t}=\frac{1}{\beta C_{m}}\left\{\nabla \cdot \left(D_{i}\nabla \left(V_{m}+\varphi_{e}\right)\right)-\beta \left(I_{\mathrm{ion}}+I_{s}\right)\right\},$$ (1)

$$\nabla \cdot \left(\left(D_{i}+D_{e}\right)\nabla \varphi_{e}\right)=-\nabla \cdot \left(D_{i}\nabla V_{m}\right),$$ (2)

where V_m_ is the membrane potential, β is the membrane surface-to-volume ratio, C_m_ is the membrane capacitance, D_i_ and D_e_ are intracellular and extracellular conductivity tensors, respectively, φ_e_ is the extracellular potential, I_ion_ is the ionic current, and I_s_ the stimulation current. I_ion_ is obtained by cellular electrophysiology models, such as those described above. Eqs. (1) and (2) can be solved simultaneously to obtain spatiotemporal distributions of both transmembrane and extracellular potentials in the heart. The numerical methods of solving those equations can be found in the literature.^14–17^ The monodomain model is represented by Eq. (1) alone with no extracellular potential in the equation. The distribution of the membrane potential in the heart as a function of time obtained by solving the above equations numerically provides the pattern of electrical wave propagation in the heart. While bidomain model describes the electrophysiological change of the heart in more detail and consequently provides more accurate predictions of the electrical wave propagations in the heart, the computation takes much longer time compared to the monodomain model. The monodomain model is often used instead of the bidomain model to save computational time.^14,17^ The mesh on which the equations are solved numerically is obtained by constructing the geometry of the heart by segmentation of medical images and generation of an appropriate type of grid. One-dimensional (1D) models are constructed by linking many cellular elements consisting of endocardial, mid-myocardial, and epicardial cells. Each cellular element is modeled using an electrophysiological model of ventricular cells, such as the ORd model, and can be depolarized once an electrical signal arrives. Propagation of the electrical signal is simulated by solving a reaction–diffusion equation^18^ following electrical stimulation at one end of the 1D model. To obtain a valid morphology of ECG, the ratio of the thicknesses of the endocardial, mid-myocardial, and epicardial layers should be appropriately determined because the positive T wave results from the relatively short APD of the epicardial layer. A 2D model is a spatial extension of a 1D model, with the very important advantage that it allows examination of the pattern of cardiac wave propagation, such as reentry. The simulation is performed by solving a 2D reaction–diffusion equation.^18^ Various S1–S2 protocols can be applied to initiate reentry. Simulated ECG can also be obtained. Three-dimensional (3D) biophysical models of the heart can be used to evaluate the proarrhythmic potential of drugs by simulating ECG from the heart model. Torso models can also be included in these model systems. ECG simulated from a 3D model is closer to the real ECG than pseudo-ECG from 1D or 2D models. The 3D heart model can also be used to examine the pattern of cardiac wave propagation in the heart model under the effects of drugs.

**FIG. 2.**
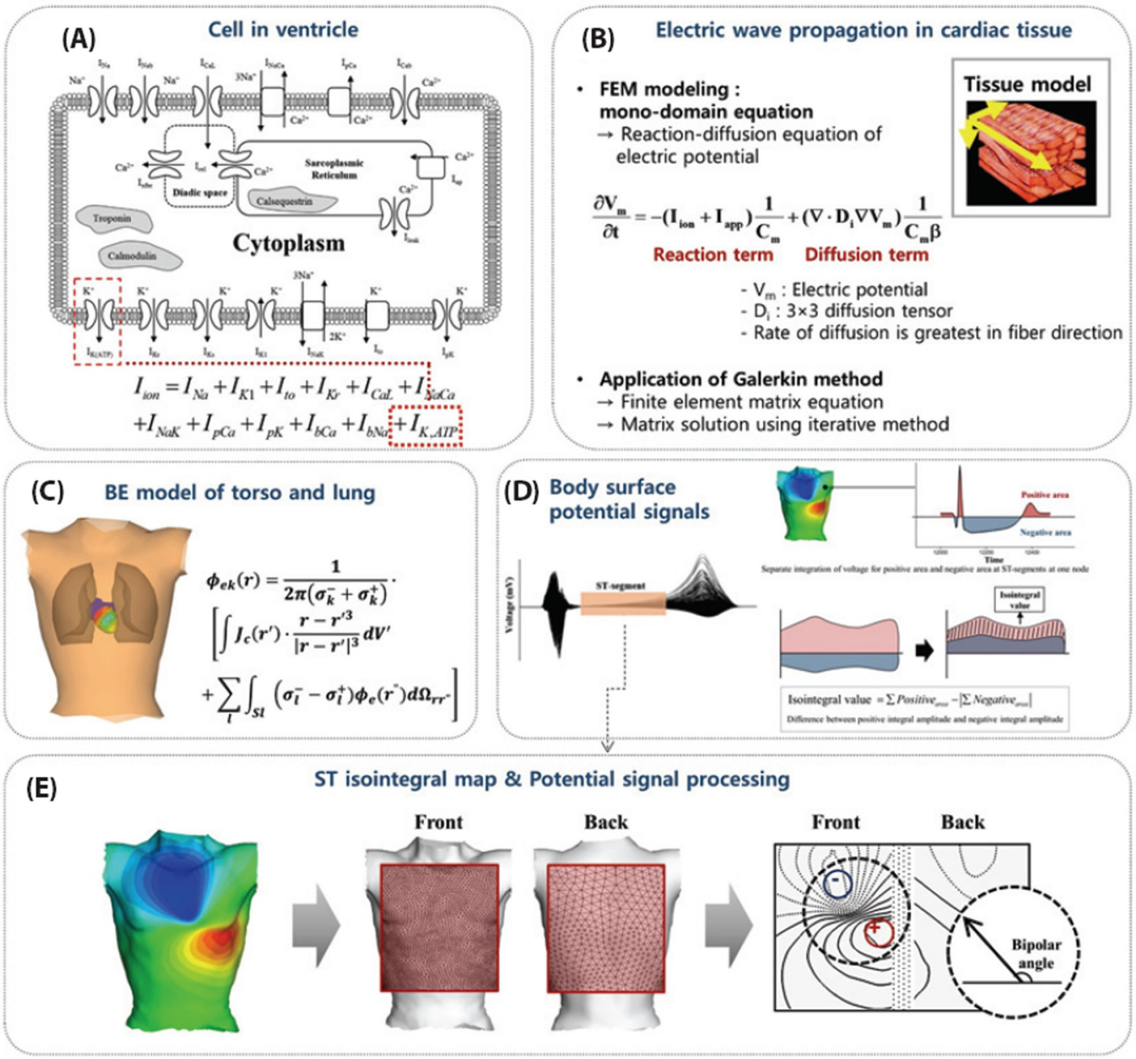
Simulation of cardiac electrophysiology. (a) An example of a cell model. (b) Simulation of electrical wave propagation. (c) Electrical potential calculation. (d) Body surface potential. (e) Electrical potential signal processing. Reproduced with permission from Ryu *et al.*, Korean J. Physiol. Pharmacol. **23**(1), 71–79 (2019). Copyright 2019 Authors, licensed under a Creative Commons Attribution Non-Commercial License.

The heart model for drug safety evaluation can range from a ventricular model to a whole heart model combined with a torso model depending on the type of data needed (Fig. 2).^19,20^ The ventricular model generates the QRS complex and the T wave without the P wave in the ECG. If the P wave needs to be checked, the atrial or whole heart model should be used. The inclusion of a very detailed model of the sinoatrial node in the atrial model would provide the data on the effects of drugs on the automaticity of electrical signal generation in the heart.^21–23^ The incorporation of the Purkinje fibers in the ventricular model would be indispensable because the morphology of the ECG is closely related to the direction of the electrical signal propagation guided by the Purkinje fibers. There are a number of models of Purkinje fibers^24–26^ such as the one developed by Cardone-Noott *et al.*^26^ in which the Purkinje fibers are emulated by including root nodes and a fast activation endocardial layer. The ECG obtained by placing an electrode at a distance from a model without the torso model is a pseudo-ECG which should be interpreted accordingly.

Ventricular arrhythmia (VA) is an irregular propagation of the electrical signal in the ventricles, which can lead to sudden cardiac death. Simulation of the electrical wave propagation in the ventricles can be used to clarify the mechanisms of VA and develop new treatment strategies. Lim *et al.*^27^ simulated electrical wave propagation in the ventricles and developed a heart–torso model capable of generating body surface potential maps and ECG waveforms. They determined the optimal placement of a bipolar mini-ECG for ubiquitous healthcare. Deng *et al.*^28^ conducted simulations of ventricular tachycardia (VT) using ventricular models reconstructed from late gadolinium-enhanced magnetic resonance imaging (MRI). They examined the effects of the electrophysiological parameters on VT and predicted ablation targets in the heart model.

## III. PROARRHYTHMIC RISK EVALUATION OF DRUGS

### A. *In vitro* experiments to determine the effects of drugs on ion channels

In the evaluation of the proarrhythmic risk of drugs using *in silico* models, the effects of drugs are incorporated into the models by partly blocking appropriate ion channels. The extent of ion channel blockade for each drug can be determined using *in vitro* experiments. The CiPA initiative included *in vitro* experiments to determine the effects of drugs on ion channels.^1,2^ They observed that hERG, late sodium, and L-type calcium currents were the ones where the block was higher, and that blockade of late sodium and/or calcium currents reduced the risk of TdP in the presence of hERG block for low-risk drugs.^2^ The experiments were also conducted using high-throughput automated patch clamp techniques.^2^ In the case of the hERG channel, it was observed that the half-maximal inhibitory concentration (IC_50_) is not alone sufficient to characterize hERG block and temperature, time, voltage, and states also significantly affect the interactions between drugs and the hERG channel.^29^ Moreover, the IC_50_ value could differ depending on the measurement protocol.^30^ Gomis-Tena *et al.*^30^ proposed a three-protocol IC_50_ assay to estimate the potency to block I_Kr_ *in vitro*. Li *et al.*^31,32^ developed a model of dynamic interactions between drugs and the hERG channel, incorporated the dynamic model into the ORd model, and predicted the torsadogenic risk of drugs. There have also been experiments that showed the effects of drugs on the blockade of multiple ion channels.^33,34^ It was found that the effect of the blockade of the hERG channel can be offset by a concomitant block of other currents.^33,34^

### B. Single cell simulations

*In silico* models of cellular electrophysiology can be used to examine the changes in action potential (AP) biomarkers such as the action potential duration (APD), AP peak, resting membrane potential, and calcium transient under the effects of drugs.^35–40^ The change in the APD is directly reflected in the morphology of ECG. The CiPA initiative included the prediction of APD change using *in silico* cell models.^1^ The cell models described in Sec. II can be used to examine the effects of drugs on the change of AP morphology and duration by incorporating an experimentally measured ion channel blockade. Wilhelms *et al.*^41^ examined the effects of amiodarone and cisapride on cellular APD, the amplitude of AP, and resting membrane potential for healthy and ischemic cells using the TT06 model. They tested low and high concentrations of the drugs. Luo *et al.*^18,42^ examined the effects of amiodarone, quinidine, disopyramide, and E-4031 on AP morphology using the TT06 model. They replaced the I_Kr_ model equation with a Markov change formulation and incorporated I_NaL_ from the ORd model. They modified the parameters of I_K1_ to incorporate the experimentally observed kinetic properties of the channel, and examined the transmural AP heterogeneity by simulating endocardial, mid-myocardial, and epicardial cells. Kubo *et al.*^43^ examined the effects of dofetilide on cellular AP in models of failing and non-failing hearts based on the ORd model. They included the protein binding rate into their model, and observed EAD at a dofetilide concentration of 100 nM in the non-failing heart model and at 25 nM in the failing heart model. Romero *et al.*^44^ examined the effects of 84 drugs on the AP of endocardial, mid-myocardial, and epicardial cells using the ORd model. Chang *et al.*^45^ performed an uncertainty quantification on the variability in pharmacology data and evaluated the robustness of TdP risk separation by qNet, an *in silico* TdP risk metric that they proposed. Lancaster and Sobie^46^ constructed classifiers that can assess the arrhythmogenicity of drugs combining simulations of drug effects with statistical analysis and machine learning. Paci *et al.*^47^ simulated the effects of mexiletine and ranolazine on the APD of LQT3 human induced pluripotent stem cell-derived cardiomyocytes (hiPSC-CMs) using populations of *in silico* hiPSC-CM models. These studies show that specific ion channel models are sometimes adopted from different cell models and even replaced with different type of models such as a Markov model. Even with the same model formulations, the model parameters are sometimes modified using additional experimental data. Appropriate modifications to the cell models depending on the type of problem would increase the accuracy of the model prediction.

### C. One-dimensional simulations

A very important advantage of using a 1D model is that a virtual ECG can be obtained despite the very simple structure of the model by placing a virtual electrode at a distance from the 1D model and solving the governing equation of electrical potential.^18^ O'Hara *et al.*^8^ obtained pseudo-ECG from a 1D model based on their ORd model and verified that the T-wave was upright and rate-dependent. Moreno *et al.*^48^ performed a 1D simulation under the effects of ranolazine and reported marked prolongation of QTc, which was not consistent with clinical data. When they included weaker ranolazine metabolite inhibition of I_Kr_ in their model, the QTc prolongation was consistent with clinical data. Wilhelms *et al.*^41^ examined the effective refractory period, slope of APD_90_, conduction velocity, and wavelength restitution curves using a 1D model under the effects of amiodarone and cisapride and compared these parameters under ischemic and healthy conditions. Luo *et al.*^18,42^ examined the pseudo-ECG from a 1-D model under the effects of E-4031, disopyramide, quinidine, and amiodarone. They used an I_Kr_ ratio of 1.6:1:1 in the epicardial, mid-myocardial, and endocardial cells based on the experimental study,^49^ which generated a positive T-wave amplitude. Patel *et al.*^50^ examined the effects of citalopram and its primary and secondary metabolites on QT interval prolongation using the pseudo-ECG obtained from a 1D model. They examined the effects using unbound or total plasma as the operating drug concentration. Romero *et al.*^44^ examined the effects of 84 drugs on the QT interval using pseudo-ECG obtained from a 1D model, and proposed a new index for discriminating torsadogenic compounds, which was defined as the ratio of the drug concentrations producing 10% prolongation of the cellular endocardial, midmyocardial, and epicardial APDs and the QT interval, over the maximum effective free therapeutic plasma concentration. Polak *et al.*^51^ developed a cardiac risk algorithm using pseudo-ECG obtained from a 1D model. They simulated increasing concentrations of 96 reference compounds and used multiple machine learning techniques to develop an algorithm that can classify drugs according to TdP risk.^51^ They tested machine learning algorithms including decision trees, random forests, and support vector machines, and the model using alternating decision tree was found to be the best in TdP risk classification. The input variables include the time gap between the end of electric and mechanical systole, and the index of cardiac electrophysiological balance (=QT/QRS) obtained from pseudo-ECG of a 1D model. The output is the probability of TdP risk. The algorithm correctly classified 89% of reference compounds and 10 out of 12 validation compounds. Loewe *et al.*^52^ simulated the effects of amiodarone and dronedarone on the occurrence of atrial fibrillation (AF) by adapting the Courtemanche–Ramirez–Nattel model to represent chronic AF and hERG mutations. They observed that there are significant differences in the arrhythmia scores that they computed between the two drugs.

### D. Two-dimensional simulations

Kubo *et al.*^43^ constructed a transmural 2D model consisting of endocardial, mid-myocardial, and epicardial layers. Their model included a model of fiber orientation to achieve a transmural difference in conduction velocity. They amplified I_Na_ of the ORd model to adjust the conduction velocity to clinical values. They applied a stimulus to a section of the endocardial border and observed arrhythmia under the effects of dofetilide at its supratherapeutic proarrhythmic concentration. They also examined simulated ECG under the effects of six drugs and compared the results with those of a prospective clinical study. Luo *et al.*^18,42^ also constructed a transmural 2D model consisting of endocardial, mid-myocardial, and epicardial layers. A reentrant wave was initiated with an S1–S2 protocol. An S1 stimulus was applied to the side of the endocardial layer and an S2 stimulus was applied to the junction region of the mid-myocardial and epicardial layers to generate unidirectional wave propagation. The effects of quinidine, disopyramide, E-4031, and amiodarone on the dynamic behaviors of the wave were evaluated under conditions of short QT syndromes.

### E. Three-dimensional simulations

**FIG. 3.**
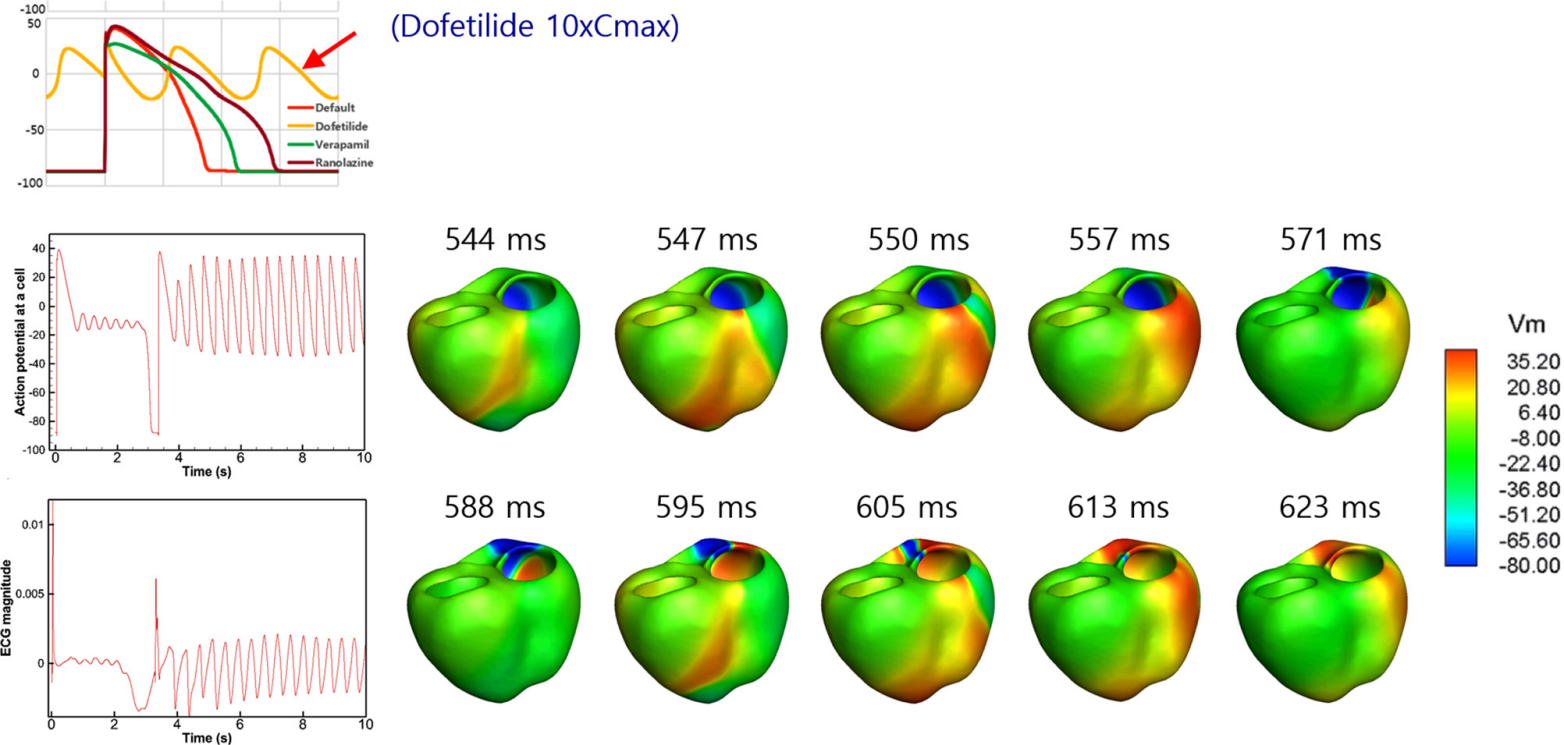
3D model for evaluating the drug effects. Action potentials from a single cell and 3D model under the effects of dofetilide are shown. Action potential distributions in the ventricular model are also shown. Reproduced with permission from Hwang *et al.*, Front. Physiol. **10**, 1139 (2019). Copyright 2019 Authors, licensed under a Creative Commons Attribution License.

**FIG. 4.**
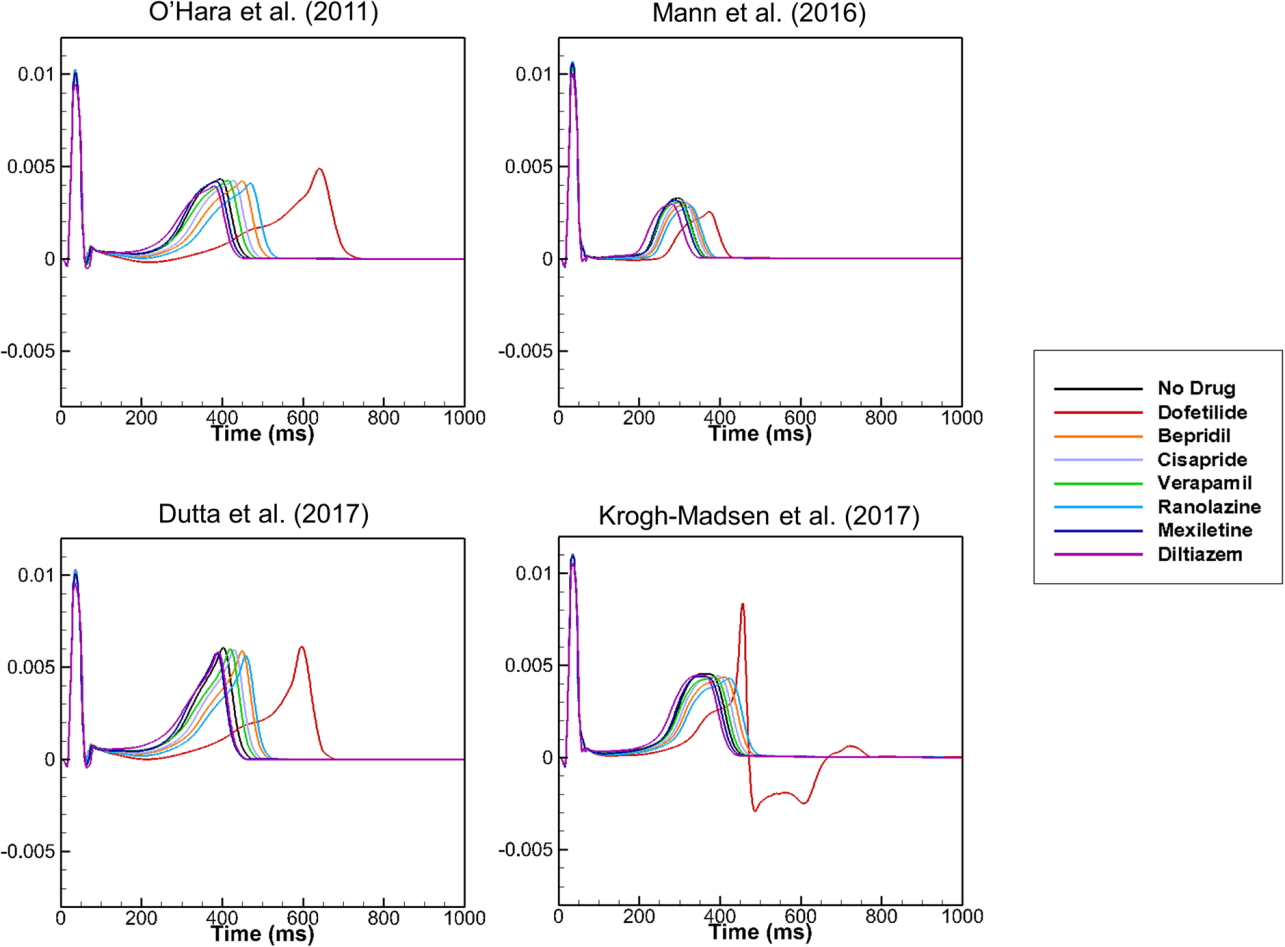
Effects of cell models on ECG. Simulated ECGs are shown for different cell models under the effects of seven drugs. Reproduced with permission from Hwang *et al.*, Front. Physiol. **10**, 1139 (2019). Copyright 2019 Authors, licensed under a Creative Commons Attribution License.

There have been studies in which an *in silico* heart model was developed and simulations were performed to test the virtual heart as a platform for screening drug toxicity including the effects of drugs on the short QT syndrome.^53–56^ Dux-Santoy *et al.*^57^ examined the effects of dofetilide on the cardiac conduction system (CCS) using a patient-specific ventricular model and observed the differences in the distribution of APD with and without the CCS. Wilhelms *et al.*^41^ developed a 3D model of the ventricles using MRI images of a healthy volunteer. The fiber orientation was generated using a rule-based method and the His-Purkinje system was mimicked by an endocardial stimulation profile. The endocardial and mid-myocardial tissues each occupied 40% of the ventricular wall with the epicardial tissue occupying 20%. The conductivity at the apex was twice that at the base. They examined the effects of amiodarone and cisapride on the ECG and conduction-related properties, such as the conduction velocity and wavelength, using the 3D model. Zemzemi *et al.*^58,59^ developed a 3D anatomical finite-element mesh of the human body from human anatomical data. They included realistic fiber orientation in the model using a rule-based method. The electrical activity was simulated using bidomain equations, and the heart–torso interface was assumed to be a perfect conductor. Different conductivity tensor values were used in the heart and different parts of the body. The endocardium surface was progressively activated from the apex to the base to mimic Purkinje network activation. They examined the effects of blocking I_Kr_, I_Na_, and I_CaL_ on ECG using the 3D model. Costabal *et al.*^60^ developed a 3D model of the human heart using the ORd model for ventricular cells and the Stewart model^25^ for Purkinje cells, which has a feature of automaticity. The ventricular wall consisted of 20% endocardial cells, 30% mid-wall cells, and 50% epicardial cells, which ensured positive T waves. The Purkinje network was generated as a fractal tree that grew from four locations. They examined the effects of dofetilide on ECG using the 3D model. Okada *et al.*^61,62^ created a 3D model of the heart and torso from the multi-detector computed tomography (CT) images of a healthy adult. They replaced the model equations for the m gate of the Na channel with those of the TT04 model to reproduce the physiological conduction velocity. The adjustments of the sites of interaction between the Purkinje network and myocardium were needed to reproduce the normal QRS morphology. They used bidomain equations to obtain the propagation of excitation. They created a five-dimensional hazard map with coordinates representing the percentage of the block of I_Kr_, I_Na_, I_NaL_, I_CaL_, and I_Ks_ using simulated ECGs. They compared the arrhythmogenic risk evaluation based on the hazard map with those reported in the literature, and found that the JTpeak was a superior index of arrhythmogenicity compared to the QT interval. We also developed a 3D model of the human heart and torso for the evaluation of drug safety (Fig. 3).^63^ We examined the effects of seven drugs with high, intermediate, and low proarrhythmic risks on ECG. We tested three optimized cell models based on the ORd model as well as the ORd model, and examined the effects of the cell models on ECG under the effects of the seven drugs (Fig. 4). A significant increase in JTpeak interval was observed under the effects of verapamil, which is a safe drug, using the ORd model although, clinically, safe drugs did not prolong the JTpeak interval. Simulation using the cell model optimized by Mann *et al.*,^10^ however, resulted in negligible prolongation of the JTpeak interval under the effects of verapamil. The cell models developed and validated using the data obtained from cell-level experiments would require adjustments using ECG data when the model is incorporated in the heart and torso model. Recently, Levrero-Florencio *et al.*^64^ developed a human-based physiologically detailed, and fully coupled ventricular electromechanical model, and performed a high performance computing study on the sensitivity of mechanical biomarkers to key model parameters.

### F. Models of machine learning/statistical analysis

There have been studies using machine learning (ML)/statistical analysis (SA) to classify the TdP risk of drugs. The model of ML/SA can be trained using the data of drugs with a known TdP risk. After training, the ML/SA model can classify new drugs into TdP risk categories. The input data for the training of the ML/SA model can be either the direct features^33^ such as the extents of drug-induced block of ion channels or derived features^46^ such as the output from *in silico* simulations using biophysical models in the form of mathematical equations. The models that can be used for ML/SA include logistic regression,^33,65^ Gaussian process regression,^66^ support vector machine,^46,65^ and neural network models.^65^ Parikh *et al.*^65^ proposed a two-step classifier for TdP risk stratification and found that their classifiers based on direct features provided identical performance to those based on derived features as input data. The models of ML/SA can be an alternative approach to very complex 3D biophysical models for TdP risk classification.

## IV. FUTURE DIRECTIONS

**FIG. 5.**
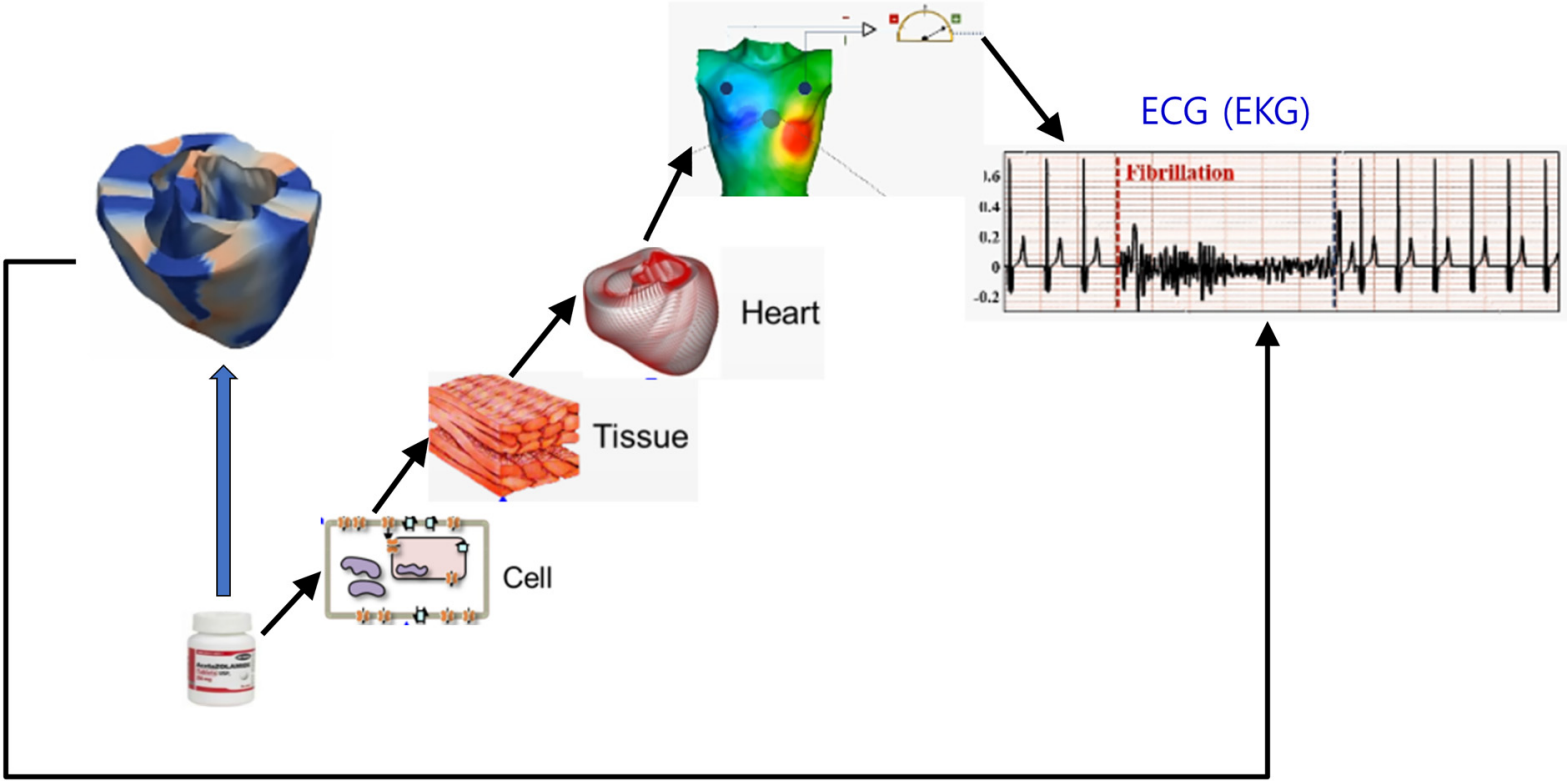
*In silico* test process for drug safety evaluation. *In silico* models from single cell to 3D models to test drug effects are shown.

The CiPA initiative included *in silico* single cell simulation in drug safety evaluation. Even though it would be ideal if a system of 3D models of the heart and torso could be used to test the proarrhythmic potential of drugs (Fig. 5), the computation time is currently prohibitive and supercomputers would be needed, which is impractical for pharmaceutical companies. One way of reducing computation time at a relatively low cost would be parallel computing using the graphics processing unit (GPU) because GPU has generally a much larger number of cores than the central processing unit (CPU) at the same price. Table I lists the currently available 3D heart models and solvers for drug safety simulations.^26,41,58,60,61,67–69^ Although multiple 3D models have been developed and studied by different groups, further validation against clinical data is needed. Recently, the JTpeak has been proposed as a more reliable index of the proarrhythmic potential, based on clinical data. As demonstrated in our study, different 3D models incorporating different cell models resulted in different changes in the JTpeak interval. Validation of the cell models is critical to improve the accuracy of the 3D models. The accuracy of fiber orientation, the Purkinje network, and distribution of fibrosis would also affect ECG under the effects of drugs. Mincholé *et al.*^70^ showed that variabilities in ventricular and torso anatomies among patients affected the ECG QRS complex. A methodology to include patient-to-patient anatomical and electrophysiological variabilities in the 3D model would be ideal. One way of incorporating these variabilities is to construct a virtual population model of the heart. There have been studies in which inter-subject variability was included in the simulation of cardiac cellular electrophysiology using a population model.^35,71,72^ Recently, Zhou *et al.*^73^ found the minimum set of ion channels required for reliable TdP risk predictions, and the effects of the variations of IC_50_ and Hill coefficient values on the accuracy of *in silico* predictions of TdP risk using a population of human ventricular cell models. Extending this methodology to a 3D model by considering the anatomical variability of the heart and torso as well would provide simulated ECGs including inter-subject variability. By assuming appropriate ranges of variations in the anatomical and electrophysiological properties of the heart based on the available data, a population of heart models with different properties can be constructed and simulations can be performed on the virtual population. The simulation results will be statistical rather than deterministic for a patient. The population modeling approaches were discussed at a Cardiac Safety Research Consortium/Health and Environmental Sciences Institute/U.S. Food and Drug Administration-sponsored meeting as a future direction.^74^ As also discussed in the meeting, novel methods to obtain the covariance of physiological parameters may need to be developed, and uncertainty quantification may need to be incorporated in inter-subject variability.^74^

**TABLE I.** List of available 3D heart models and solvers for drug safety simulations.

Reference	Model	Solver
Wilhelm *et al.*^41^	In-house	acCELLerate (https://www.ibt.kit.edu/acCELLerate.php)
Zemzemi *et al.*^58^	In-house	Chaste (https://www.cs.ox.ac.uk/chaste/)
Dutta *et al.*^67^		
Cardone-Noott *et al.*^26^		
Martinez-Navarro *et al.*^68^		
Okada *et al.*^61^	UT-heart (http://ut-heart.com/index.html)	In-house
Sahli Costabal *et al.*^60^	The living heart (Baillargeon *et al.*, 2014)	Abaqus (Dassault Systémes)
Cranford *et al.*^69^	In-house	Cardioid (https://github.com/LLNL/cardioid)

## AUTHORS' CONTRIBUTIONS

Minki Hwang and Chul-Hyun Lim wrote the draft of the manuscript. Chae Hun Leem and Eun Bo Shim reviewed and edited the manuscript.
